# The transcriptional regulation effects of histidine, isoleucine and glutamate on free exopolysaccharide biosynthesis of *Streptococcus thermophilus* 937

**DOI:** 10.3389/fmicb.2024.1476940

**Published:** 2025-01-08

**Authors:** Yunchao Wa, Xia Zhao, Chenchen Zhang, Hengxian Qu, Dawei Chen, Xia Chen, Yujun Huang, Ruixia Gu

**Affiliations:** ^1^Key Lab of Dairy Biotechnology and Safety Control, Yangzhou University, Yangzhou, Jiangsu, China; ^2^College of Food Science and Engineering, Yangzhou University, Yangzhou, Jiangsu, China

**Keywords:** *Streptococcus thermophilus*, amino acid, exopolysaccharide, transcriptomics, quantitative PCR

## Abstract

**Introduction:**

The free exopolysaccharide (f-EPS) produced by *Streptococcus thermophilus* is a natural texture modifier and has a variety of prebiotic activities. Our previous studies showed f-EPS production from *S. thermophilus* 937 was increased 2-fold in the presence of 15 mM of glutamate, isoleucine, and histidine in the chemically defined medium.

**Methods:**

In this study, we used transcriptomics and qPCR to further explore the specific mechanism of the enhanced effect of 3 amino acids on the f-EPS biosynthesis of *S. thermophilus* 937.

**Results:**

The mRNA-seq analysis and targeted pathway analysis indicated that genes associated with histidine/valine/leucine/ isoleucine/phenylalanine/tyrosine/tryptophan synthesis, galactose metabolism, purine metabolism and quorum sensing in *S. thermophilus* 937 were significantly upregulated under increasing concentrations of histidine, isoleucine and glutamate in chemically defined medium (CDM). qPCR results showed that the significant upregulation of galactose metabolism- and nucleotide sugar synthesis-related genes was attributed to the increase in glutamate concentration, and glutamate could induce the expression of *galR*. The upregulation of *epsA*, *epsB*, and *epsD* transcript levels was caused by the increase in histidine concentration. The upregulation of transcript levels of genes related to phenylalanine/tyrosine/tryptophan/histidine/ valine/leucine/isoleucine synthesis was caused by the increase in isoleucine concentrations.

**Discussion:**

This indicates that 3 amino acids have different mechanisms for enhancing the biosynthesis of f-EPS in *S. thermophilus* 937.

## Introduction

*Streptococcus thermophilus* is one of the most important lactic acid bacteria used in the dairy industry, and it is a widely used dairy starter to produce yogurt and cheese (Iyer et al., 2010). Most *S. thermophilus* can produce free exopolysaccharides (f-EPS) during the exponential growth period. The f-EPS produced *in situ* is a natural texture modifier that can improve the texture, viscosity, and water holding capacity of fermented milk (Li et al., 2014). At the same time, f-EPS also have a variety of bioactivities, such as antitumor and anti-inflammatory activities (Chen et al., 2019; Mizuno et al., 2020).

In a previous study, we examined the impact of 20 amino acids on the growth metabolism and f-EPS production of *S. thermophilus* 937 in a chemically defined medium (CDM) (Wa et al., 2023). We observed that elevating the concentrations of histidine, isoleucine, and glutamate, either individually or in combination, to 15 mM, substantially boosted the f-EPS production by *S. thermophilus* 937. The f-EPS production reached its peak when 15 mM histidine, isoleucine, and glutamate were present in the medium, nearly 2-folds compared to the control group (Wa et al., 2022; Wa et al., 2023). The 3 amino acids may promote f-EPS production by maintaining cell viability, promoting nucleotide sugar synthesis, and upregulating *epsA and epsB* transcript levels (Wa et al., 2022). In *S. thermophilus*, amino acids play a major role in regulating intracellular pH, generating energy, providing the biosynthetic precursors for the synthesis of nutrients, producing aromatic compounds, and resisting stress (Fernandez and Zuniga, 2006). Glutamate in the presence of H^+^ may produce GABA (*γ*-aminobutyric acid) by glutamate decarboxylases to increase intracellular pH (Papadimitriou et al., 2016). Histidine betaine and ergometrine (2-mercapto-histidine betaine) produced by histidine metabolism are usually used by strains of *S. thermophilus* to maintain the reducing environment (Fernandez and Zuniga, 2006). The synthesis and metabolism of isoleucine requires the participation of pyruvate, so it will directly affect the central metabolism of the bacterium, which is of great significance for maintaining the cell viability and intracellular pH (Garault et al., 2000). There are few reports about the effects of amino acid metabolism on nucleotide sugar synthesis and the transcript levels of genes in the *eps* gene cluster (Wu et al., 2022). Only studies have shown that the flux of the GalU catalytic reaction is related to the nitrogen demand of *S. thermophilus* (Svensson et al., 2007), and the reaction substrate uridine triphosphate (UTP) of GalU is involved in many nitrogen regulation systems of *S. thermophilus* (Merrick and Edwards, 1995). Our Previous study revealed that supplementing *S. thermophilus* 937 with 15 mM of histidine, isoleucine, or glutamic acid individually had a smaller effect on enhancing f-EPS production compared to adding all three amino acids together (Wa et al., 2023). This implies that the 3 amino acids likely play distinct roles in boosting f-EPS production, leading to a synergistic effect. Therefore, it is necessary to further explore the specific mechanism of 3 amino acids at the global transcriptional level.

Whole-genome sequencing and detailed gene annotation of *S. thermophilus* 937 have been completed, which makes transcriptome analysis possible. In this study, the effects of histidine, isoleucine and glutamate on *S. thermophilus* 937 were analyzed by transcriptomics, and identified genes with significant changes in transcript levels. Then, the effects of increasing the concentration of a single amino acid (histidine, isoleucine, or glutamate) on the transcript levels of these genes were determined by qPCR to explore the regulatory mechanism of histidine, isoleucine, and glutamate on the biosynthesis of f-EPS.

## Materials and methods

### Bacterial strain and fermentation condition

*Streptococcus thermophilus* 937 (CGMCC NO. 25410) is known to produce high levels of EPSs and was provided by Jiangsu Province Key Laboratory of Dairy Processing and Safety, Yangzhou University. The strain was stored at −80°C in M17 broth (Qingdao Hope Bio-Technology Co., Ltd., Qingdao, China) supplemented with 30% (v/v) glycerol.

The chemically defined medium (CDM) was prepared according to the optimized formula of previous study (Wa et al., 2022). There were 42 kinds of nutrients, including 1 sugar, 20 free amino acids, 9 vitamins, 4 nucleic acid bases, and a variety of inorganic salt substances (Table 1). The pH of the CDM was adjusted to 6.7 by 1 mol/L NaOH. CDM was sterilized by filtration through a 0.22 μm pore-size membrane (NALGENE Rapid-Flow, Thermo Fisher Scientific Inc., NY, USA). The concentrations of histidine, isoleucine and glutamate were increased to 15 mM based on the CDM as the CDM + HIE group. The concentration of histidine was increased to 15 mM based on the CDM as the CDM + H group. The concentration of isoleucine was increased to 15 mM based on the CDM as the CDM + I group. The concentration of glutamate was increased to 15 mM based on the CDM as the CDM + E group.

**Table 1:** Composition of the chemically defined medium.

Constituent	Concentration	Constituent	Concentration
Lactose	20 g·L^−1^ (0.0580 mM)	Ascorbic acid	0.5 g·L^−1^ (0.0283 mM)
NH_4_-citrate	0.6 g·L^−1^ (0.0078 mM)	Biotin	0.01 g·L^−1^ (0.00004 mM)
Urea	0.24 g·L^−1^ (0.0040 mM)	Calcium Pantothenate	0.001 g·L^−1^ (0.00000210 mM)
		Folic Acid	0.001 g·L^−1^ (0.00000227 mM)
L-Alanine	0.089 g·L^−1^ (1 mM)	Niacin	0.001 g·L^−1^ (0.00000812 mM)
L-Arginine	0.174 g·L^−1^ (1 mM)	Pyridoxine Hydrochloride	0.005 g·L^−1^ (0.0000243 mM)
L-Asparagine	0.132 g·L^−1^ (1 mM)	Riboflavin	0.001 g·L^−1^ (0.00000266 mM)
L-Aspartic acid	0.133 g·L^−1^ (1 mM)	Thiamine Hydrochloride	0.001 g·L^−1^ (0.00000296 mM)
L-Cysteine	0.121 g·L^−1^ (1 mM)		
L-glutamate	0.147 g·L^−1^ (1 mM)	Uracil	0.01 g·L^−1^ (0.000089 mM)
L-Glutamine	0.146 g·L^−1^ (1 mM)	Adenine	0.01 g·L^−1^ (0.000074 mM)
Glycine	0.075 g·L^−1^ (1 mM)	Guanine	0.01 g·L^−1^ (0.000066 mM)
L-Histidine	0.155 g·L^−1^ (1 mM)	Thymine	0.01 g·L^−1^ (0.000079 mM)
L-Leucine	0.131 g·L^−1^ (1 mM)		
L-Isoleucine	0.131 g·L^−1^ (1 mM)	KH_2_PO_4_	3 g·L^−1^ (0.022 mM)
L-Lysine	0.146 g·L^−1^ (1 mM)	K_2_HPO_4_	3 g·L^−1^ (0.017 mM)
L-Methionine	0.149 g·L^−1^ (1 mM)	MgCl_2_	0.2 g·L^−1^ (0.002 mM)
L-Phenylalanine	0.165 g·L^−1^ (1 mM)	CaCl_2_	0.05 g·L^−1^ (0.00045 mM)
L-Proline	0.115 g·L^−1^ (1 mM)	NaH_2_PO_4_	3.60 (30 mM)
L-Serine	0.105 g·L^−1^ (1 mM)	Na_2_HPO_4_	4.26 (30 mM)
L-Threonine	0.119 g·L^−1^ (1 mM)	Na-acetate	1 g·L^−1^ (0.0121 mM)
L-Tryptophan	0.204 g·L^−1^ (1 mM)		
L-Tyrosine	0.181 g·L^−1^ (1 mM)		
L-Valine	0.117 g·L^−1^ (1 mM)		

*Streptococcus thermophilus* 937 was statically incubated in M17 broth at 37°C until reaching the exponential phase of growth (8 h), after which the culture was centrifuged and the pellet washed with potassium phosphate buffer (PBS, 50 mM, pH 6.5). To ensure the elimination of carry-over nutrients, the culture cells were collected by centrifugation at 10,000 × *g* for 5 min and washed twice with equal volumes PBS (Letort and Juillard, 2001). Finally, the cells were resuspended in PBS, and the optical density value at 600 nm wavelength (OD_600_) was adjusted to 1.00 ± 0.05. The resuspension was inoculated (2%, v/v) with growth media and incubated at 42°C.

### Transcriptomics analysis

Due to the different nutritional components between CDM and M17 broth, *S. thermophilus* 937 reached the middle or late stages of exponential growth at 3 h or 5 h, respectively, in CDM and CDM + HIE. *S. thermophilus* 937 cultured to the end of logarithmic growth (5 h) in CDM and CDM + HIE were collected. Total RNA samples were extracted using Trizol reagent according to the manufacturer’s instructions (Invitrogen, CA, USA). The concentration and purity of RNA were detected by a Nanodrop 2000 spectrophotometer (Thermo Scientific, CA, USA). The integrity of the RNA was detected by agarose gel electrophoresis, and the RNA integrity number (RIN) value was determined by an Agilent 2100 (Thermo Scientific, CA, USA). The total amount of RNA required for single library construction was 2 μg, the concentration was ≥100 ng/μL, and the OD260/280 was between 1.8 and 2.2.

Ribosomal RNA (rRNA) depletion instead of poly (A) purification is performed by RiboCop rRNA Depletion Kit for Mixed Bacterial Samples (lexogen, USA) and then all mRNAs were broken into short (200 nt) fragments by adding fragmentation buffer firstly. Secondly double-stranded cDNA was synthesized with random hexamer primers (Illumina). When the second strand cDNA was synthesized, dUTP was incorporated in place of dTTP. Then the synthesized cDNA was subjected to end-repair, phosphorylation and ‘A’ base addition according to Illumina’s library construction protocol. RNA-seq transcriptome library was prepared following Illumina^®^ Stranded mRNA Prep, Ligation (San Diego, CA, USA) using of total RNA paired-end RNA-seq library was sequenced with the Illumina Novaseq 6000 (Illumina Inc., San Diego, CA, USA). The processing of original images to sequences, base-calling, and quality value calculations. The clean reads by removing low-quality sequences, reads with more than 10% of N bases (unknown bases) and reads containing adaptor sequences.

The data generated from Illumina platform were used for bioinformatics analysis. All of the analyses were performed using the free online platform of Majorbio Cloud Platform^1^ from Shanghai Majorbio Bio-pharm Technology Co., Ltd.

### Data processing and analysis

After sequencing, it was analyzed on the online platform of Shanghai Meiji Biomedical Technology Co., Ltd. Majorbio cloud platform (see text footnote 1). We thank the China National Center for Bioinformation (CNCB) for hosting our original transcriptome data in the Genome Sequence Archive (GSA) under the accession number CRA019170.

### Real-time quantitative PCR (qPCR) analysis of gene transcription

The cells cultured for 3 h were used to determine the transcript levels of *epsABCD*, and the cells cultured for 5 h were used to determine the transcript levels of the remaining genes. Through our preliminary research and literature investigation (Padmanabhan et al., 2018), we found that the transcription levels of *epsABCD* are significantly influenced by the bacterial growth cycle. When bacteria enter the late logarithmic phase (5 h), *epsABCD* is almost not transcribed. Therefore, we selected bacterial cells from the mid-logarithmic growth phase (3 h) as the experimental subjects to capture the most active transcription period of these genes. Total RNA from the cells was isolated using a UNlQ-10 Column Trizol Total RNA Isolation Kit (Sangon Biotech Co., Ltd., Shanghai, China) according to the manufacturer’s protocol. The cell samples from each group were pooled for RNA extraction. The primers used for the reference gene (*16s rDNA*) and target genes were designed using Oligo 7.0 software and are shown in Table 2. Quantitative real-time PCR was performed using an ABI Prism 7300 Detection System (Applied Biosystems, United States). All reactions were performed in duplicate using a kit provided by Vazyme Biotech Co., Ltd.

**Table 2:** qPCR primers.

Gene	Forward primer (5′ → 3′)	Reverse primer (5′ → 3′)
*epsA*	TAAAGACTAGAATTTGGCATC	AGGTTCTAAATTTACGGTGA
*epsB*	TGATTTCTCGAACACGGTCA	AAGAAATTCATAGTGGGCTT
*epsC*	GATTTCACGTACCAATACACC	CTAAATTGCCAGAGTCACC
*epsD*	CTAGCAAATGAAATCGCCAA	TTAAAGTGATTGCGATTAGCTC
*laci*	TCATCTCTGATCCTCGTTT	TTCATAGCCAGATTGGACA
*galU*	CTTATGGTGTAATCGCTCCT	TAGCAAGATTACTCGGTGCAT
*galE*	ATGGATGCACATAGTCACGAA	GAAACTCACCTTCTACCGAT
*galM*	GATAAATCCTGTTTGTCGCTA	TACTCTTTGAAGCCTTCGAT
*galT*	TTTGAACCTGCGAAGTAACCAG	CCTTATGCCTACTTTAACGAAC
*galK*	TTAACCAAGTCCAGGCGTTC	ATGGTAACATTCCAAACGGAT
*hisG*	CAAATTACGTCCCTTATCAGC	AAATCACAATTGCCTTGACC
*hisF*	ATATTTACCTCACCGAAGTGGA	GATGTCCCAATCATTGCGTCT
*leuB*	AACGACAAAATCTACGCCTT	TCTTTGCTAATATCCGTCCTGT
*leuC*	ACAATTCCTTGCTTATCCGAAC	GGCACAAATTGATAAACTTGCT
*ilvC*	CATCAGCCAATTTAGTTGCTT	CAAAACTTGCGTGATACAGG
*ilvB*	AACAACTGGCAAATCTACCG	CGCTTGACAGGTAATCCAA
*pelf*	TTCCAAAACAGAAAGTGGCAA	TTCTATTCACTGGTCGGGTT
*trpD*	TTCAAGGACATCTGCCGAAC	TTAACATTTCAACAACGGCTT
*16 s*	CTAACTACGTGCCAGCAGC	GGTTGAGCCACAGCCTTTA

The relative gene mRNA levels were determined using the ∆cycle threshold (∆Ct) method, and *16s rDNA* served as a reference gene. For each target gene, the ∆∆Ct values of all samples were calculated by subtracting the average ∆Ct of the CDM from the average ∆Ct of the CDM + HIE. The ∆∆Ct values were converted to fold differences by raising 2 to the power of ∆∆Ct (i.e., 2^-∆∆CT^) (Livak and Schmittgen, 2001).

### Statistical analysis

Experiments were minimally performed in biological triplicates, each with at least two technical replicates. Data were analyzed using GraphPad Prism (Version 9.0.0 for Windows, GraphPad Software, San Diego, California, USA) using a T test with two-tailed calculations and one-way ANOVA with either Dunnett’s or Tukey’s post-hoc multiple comparison test as described in the figure legend.

## Results

### RNA-seq data production and read mapping/alignment

As shown in Table 3, approximately 97.0% of raw reverse reads per sample passed filtering at a quality threshold of 20. Each sample had 3 gigabases. Among these screened pairs, the unique pairing rate of each sample exceeded 95%, and they uniquely mapped to the genome of *S. thermophilus* 937; this provides enough sequencing depth for identifying DEGs.

**Table 3:** Sequence data production from RNA-seq and sequence read mapping by Bowtie.

Sample name	Clean reads	Clean bases (bp)	Q20 (%)	Genome mapped ratio (%)	Uniq mapped reads ratio (%)
CDM_Rep1	22,963,492	3,192,846,595	97.36	99.13	95.84
CDM_Rep2	25,015,878	3,451,710,471	97.38	99.10	96.08
CDM_Rep3	23,471,184	3,259,262,880	97.32	99.12	95.84
CDM + HIE_Rep1	26,291,598	3,672,205,500	97.42	99.16	95.36
CDM + HIE _Rep2	26,326,112	3,617,223,561	97.37	99.12	96.07
CDM + HIE _Rep3	24,043,404	3,355,171,049	97.19	98.99	95.88

### RNA-seq analysis

**Figure 1:**
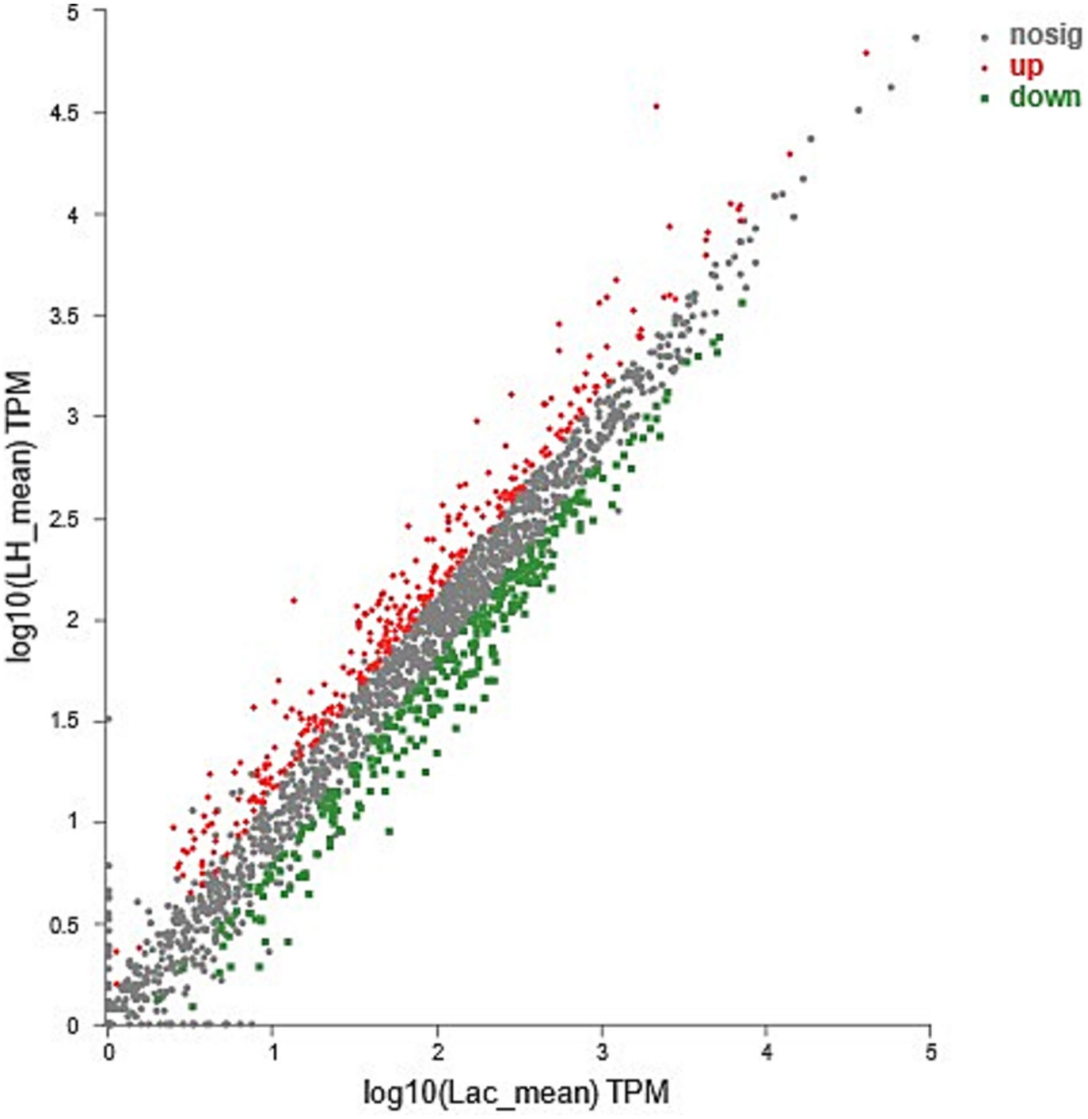
Detection of DEGs for samples from CDM + HIE compared to samples from CDM.

General presentations of the RNA-seq analysis results are shown in Figure 1 and Table 4, and the detailed expression data (log2CP and log-fold-change) are presented in Supplementary Table S1.

**Table 4:** COG annotation classification.

COG type	COG type description	Total genes	Up DEGs	Down DEGs
D	Cell cycle control, cell division, chromosome partitioning	1	0	1
M	Cell wall/membrane/envelope biogenesis	14	7	7
O	Posttranslational modification, protein turnover, chaperones	5	2	3
T	Signal transduction mechanisms	3	2	1
U	Intracellular trafficking, secretion, and vesicular transport	1	0	1
V	Defense mechanisms	7	0	7
J	Translation, ribosomal structure and biogenesis	17	6	11
K	Transcription	8	2	6
L	Replication, recombination and repair	21	3	18
E	Amino acid transport and metabolism	36	27	9
F	Nucleotide transport and metabolism	9	7	2
G	Carbohydrate transport and metabolism	9	8	1
H	Coenzyme transport and metabolism	5	2	3
I	Lipid transport and metabolism	4	1	3
P	Inorganic ion transport and metabolism	6	0	6
Q	Secondary metabolites biosynthesis, transport and catabolism	1	1	0
S	Function unknown	66	31	35

Compared with CDM, the transcript levels of 249 genes in CDM + HIE changed significantly (*p* < 0.05), of which 120 genes were significantly upregulated and 129 genes were significantly downregulated (*p* < 0.05, Figure 1).

According to the results of COG annotation (Table 4), 213 significantly differentially expressed genes were divided into 17 categories (CDM + HIE vs. CDM). Excluding category S (function unknown), the genes involved in amino acid transport and metabolism (E) as well as replication, recombination, and repair (L) were the most abundant, with 36 and 21, respectively. This was followed by 17 and 14 genes involved in translation, ribosomal structure and biogenesis (J) and cell wall/membrane/envelope biogenesis (M), respectively. In addition, there were 9 genes involved in carbohydrate metabolism (G) and nucleic acid transport metabolism (F).

### Free exopolysaccharide biosynthesis

**Figure 2:**
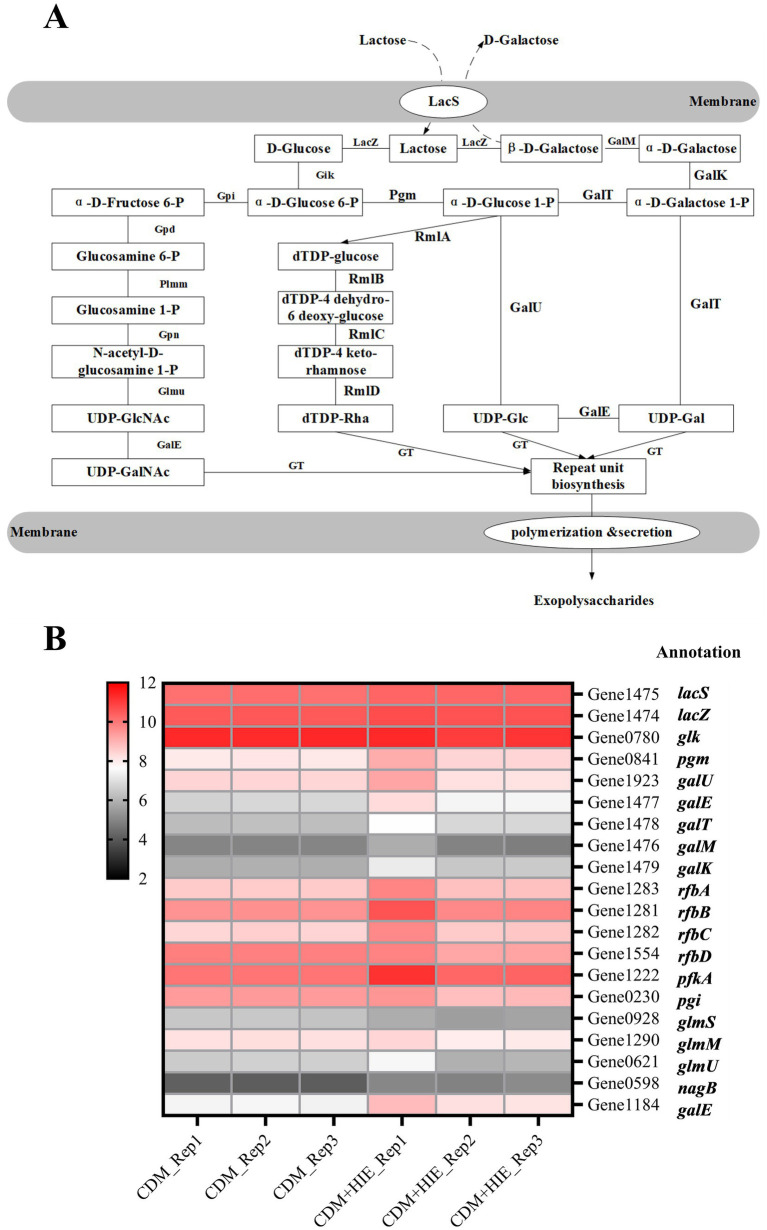
Nucleotide sugar synthesis pathway of *Streptococcus thermophilus* 937 and changes in transcript levels of related genes in CDM and CDM + HIE. **(A)** The biosynthetic pathway of nucleotide sugars. **(B)** Transcription level thermogram (log2CPM) of genes related to nucleotide sugar synthesis of *Streptococcus thermophilus* 937 in CDM and CDM + HIE.

In both CDM and CDM + HIE, lactose was used as the sole sugar source. According to the results of whole gene sequencing and gene annotation (NCBI: SAMN26293197: ST937), the sugar nucleotide synthesis pathway of *S. thermophilus* 937 with lactose as the sole sugar source was analyzed in Figure 2A. Lactose entered the cell through LacS and was enzymatically hydrolyzed into glucose and galactose by lacZ (Xiong et al., 2019). Galactose is metabolized through the Leloir pathway and transformed into UDP-Glc and UDP-Gal. Glucose was phosphorylated into glucose 6-P, then into glucose 1-P or fructose 6-P, and finally into UDP-GalNAc, dTDP-Rha and UDP-Glc (Figure 2A; Wu et al., 2022).

The transcript levels of coding genes of all enzymes involved in the sugar nucleotide synthesis pathway of *S. thermophilus* 937 in CDM and CDM + HIE were summarized in Figure 2B. The expression of *pgm*, *galE* (Gene 1,477), *galT*, *galM*, *galK*, *rfbA*, *rfbB*, *rfbC*, *pfkA*, *nagB* and *galE* (Gene 1184), in *S. thermophilus* 937 in CDM + HIE was higher than that in CDM, indicating that the addition of 3 amino acids stimulated the metabolism of galactose, transformed it into UDP-Glc and UDP-Gla, and transformed Glc 1-P to dTDP-Rha (Figure 2B). In *S. thermophilus* 937, the *galE* exists in two copy numbers within the f-EPS synthesis pathway. Existing literature primarily reports that *galE* encodes UDP-glucose 4-epimerase, an enzyme responsible for the interconversion between UDP-Glc and UDP-Gal. Due to the non-identical substrate conversion of these two copies and their genomic positioning, we hypothesize that *galE* (Gene1184) may represent a different isoform or a closely related paralog of *galE* (Gene1477). This observation underscores the genetic complexity of EPS biosynthesis in *S. thermophilus* and suggests potential functional diversification in UDP sugar metabolism.

**Figure 3:**
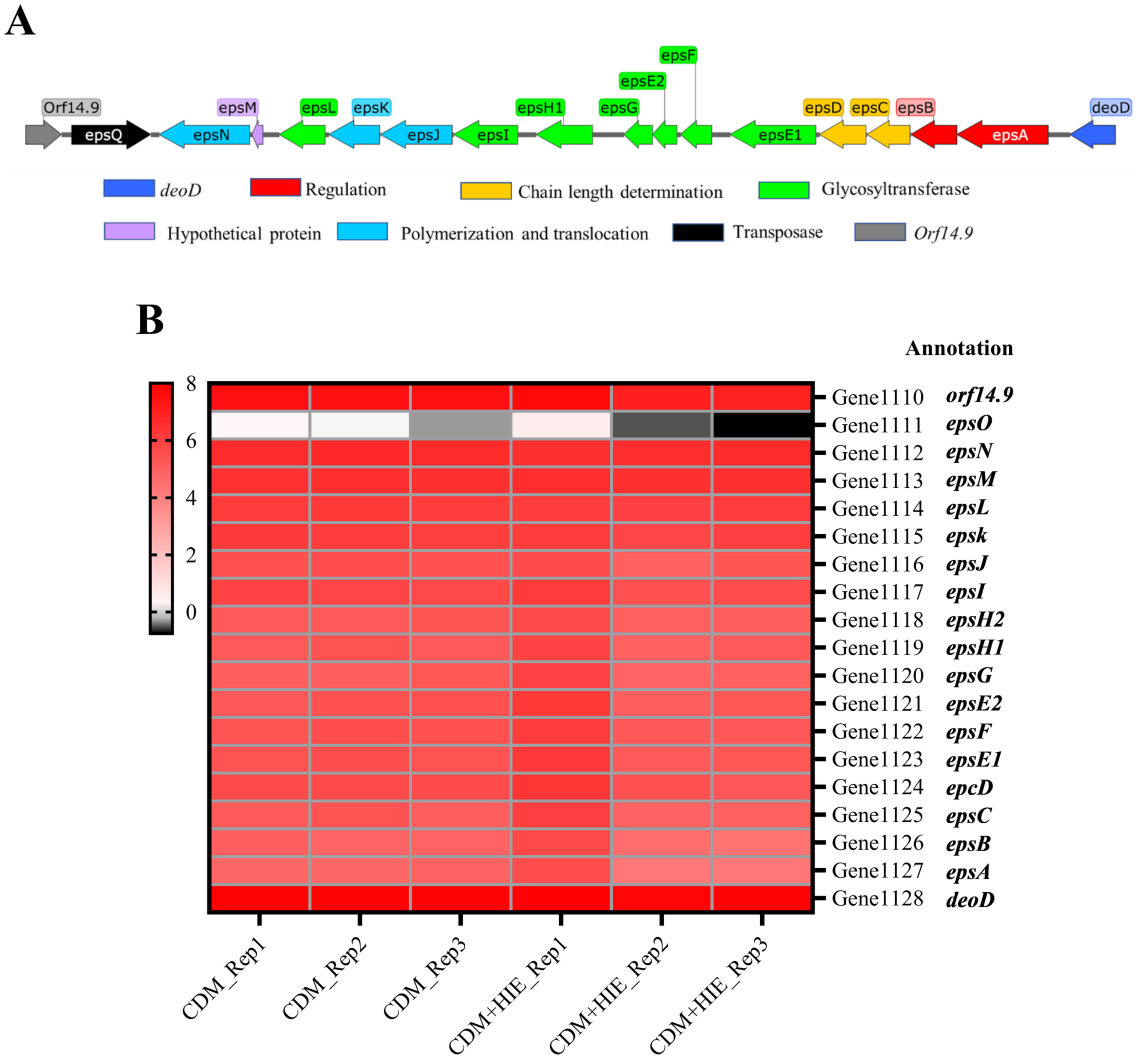
The *eps* gene cluster of *Streptococcus thermophilus* 937 and Changes in transcript levels of related genes in CDM and CDM + HIE. **(A)** The biosynthetic pathway of nucleotide sugars. **(B)** Transcription level thermogram (log2CPM) of genes related to *eps* gene cluster of *Streptococcus thermophilus* 937 in CDM and CDM + HIE.

The *eps* gene cluster of *S. thermophilus* 937 is shown in Figure 3A. The transcript levels of *eps* cluster genes of *S. thermophilus* 937 in CDM and CDM + HIE were summarized (Figure 3B). Compared with CDM, the gene transcript levels of the *eps* gene cluster of *S. thermophilus* 937 did not change significantly in CDM + HIE, which may be related to the sampling time point.

### KEGG enrichment analysis

According to the KEGG database, the functional enrichment of significantly upregulated genes (FC>2, *p*<0.05) was analyzed. Fisher’s exact test was used. When the *p* value<0.05, it was considered that the function of the KEGG pathway was significantly enriched.

**Figure 4:**
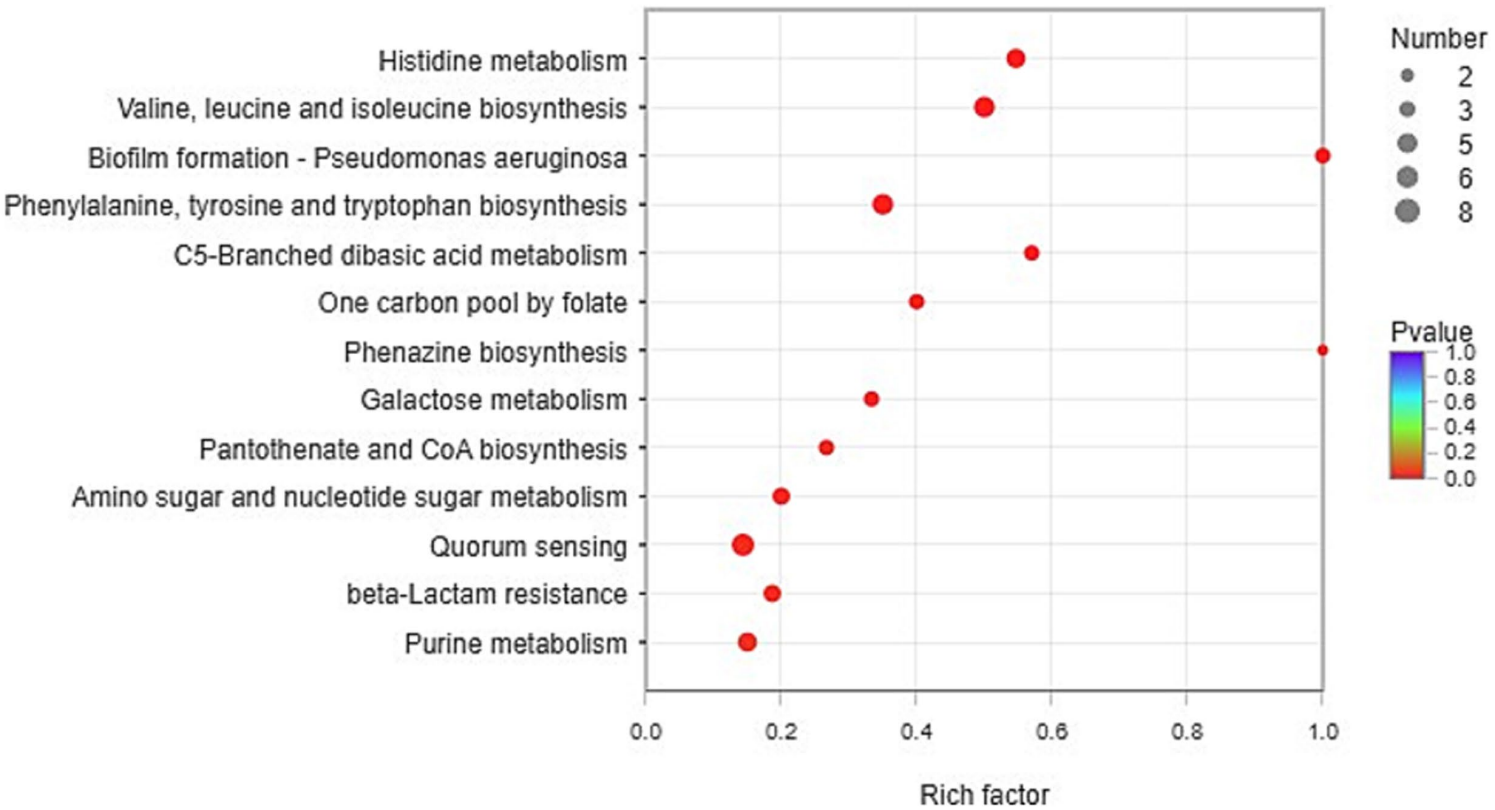
KEGG enrichment analysis (CDM vs. CDM + HIE).

The pathways of histidine metabolism, valine, leucine and isoleucine biosynthesis, biofilm formation, phenylalanine, tyrosine and tryptophan biosynthesis, C5-branched dibasic acid metabolism, one carbon pool by folate, phenazine biosynthesis, galactose metabolism, pantothenate and CoA biosynthesis, amino sugar and nucleotide sugar metabolism, quorum sensing, beta-lactam resistance, and purine metabolism were found to be significantly expressed in CDM + HIE (Figure 4).

**Figure 5:**
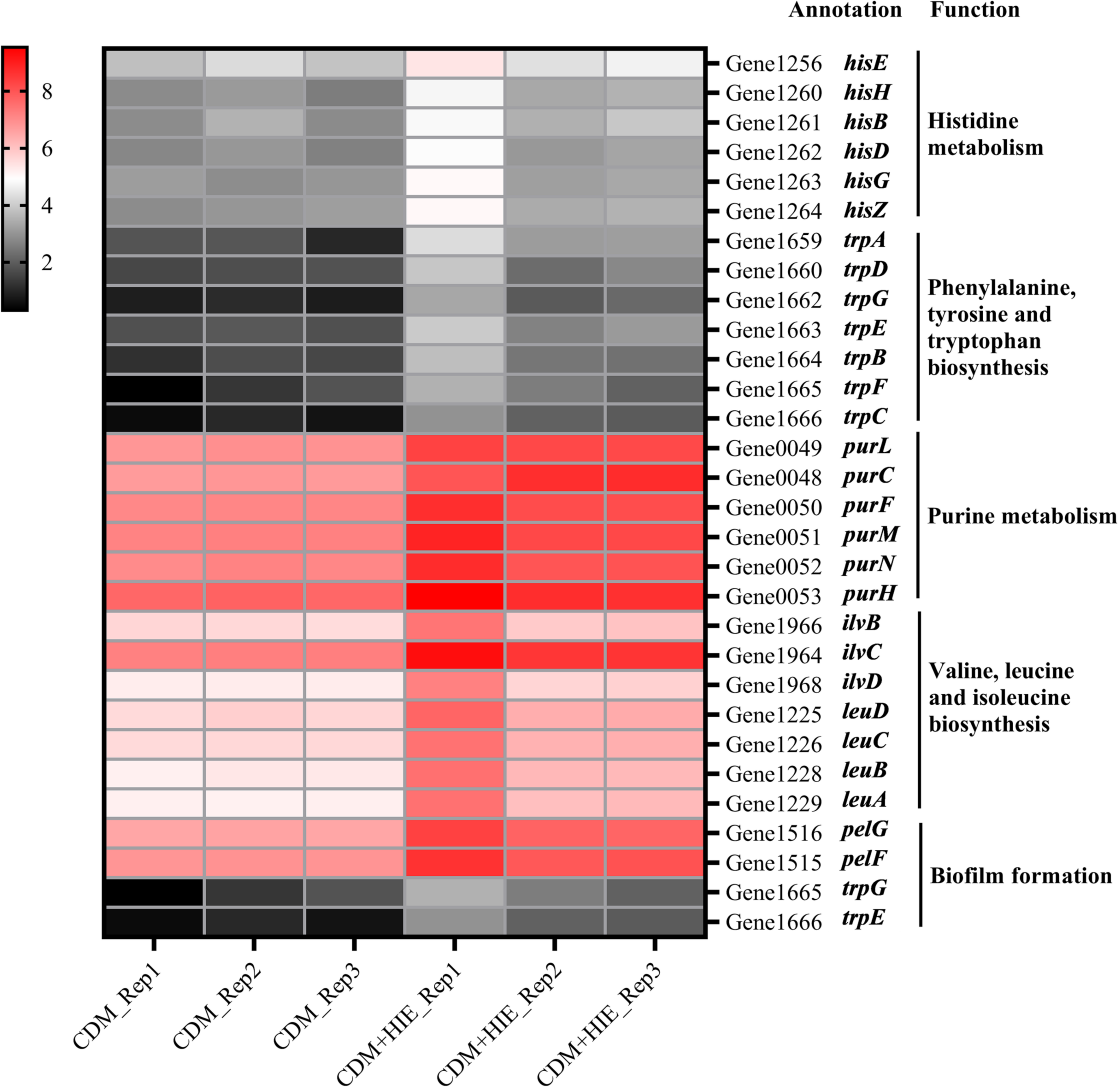
Changes in transcript levels (log2CPM) of histidine/phenylalanine/tyrosine/tryptophan/purine metabolism, valine/leucine/isoleucine biosynthesis and biofilm formation associated genes in *Streptococcus thermophilus* 937 in CDM and CDM + HIE.

The genes with significantly upregulated transcript levels of histidine metabolism, phenylalanine/tyrosine/tryptophan biosynthesis, and purine metabolism were presented in Figure 5 and Supplementary Table S1. According to the KEGG, *hisE*, *hisH*, *hisB*, *hisD*, *hisG*, and *hisZ* were the genes of the Phosphoribosyl pyrophosphate (PRPP) synthesized histidine pathway; *trpA*, *trpD*, *trpG*, *trpE*, *trpB*, *trpF*, and *trpC* were the genes of the PRPP synthesized indole or tryptophan pathway; *purL*, *purC*, *purF*, *purM*, *purN*, and *purH* were the genes of the PRPP synthesized aminoimidazole ribopeptide (AIR) pathway. This indicates that increasing the concentration of histidine, isoleucine and glutamate to 15 mM in CDM can significantly up regulate the genes in the pathway of PRPP used to synthesize histidine, indole, tryptophan or AIR in *S. thermophilus* 937.

The genes with significantly upregulated transcript levels of valine/isoleucine/leucine biosynthesis and biofilm formation were presented in Figure 5 and Supplementary Table S1. According to the KEGG, *ilvB*, *lvC*, *ilvD*, *leuD*, *leuC*, *leuB*, and *leuA* were the genes of the pyruvic synthesized valine, isoleucine and leucine pathway. Among them, *ilvB*, *lvC*, and *ilvD* were genes in the pyruvate-synthesized 3-methyl-2-oxobutyrate pathway in the pantothenic acid and coenzyme A biosynthesis pathway, and *ilvB is* a gene in the pyruvate-synthesized (s)-2-acetolactate pathway in the C5 branched chain dicarboxylic acid metabolism pathway. *pelF* and *pelG* encode polysaccharide biosynthetic proteins and were significantly upregulated (*p* < 0.05, Figure 5) in the biofilm formation pathway.

### Effects of glutamate on the transcript levels of genes related to galactose metabolism and nucleotide sugar synthesis

**Figure 6:**
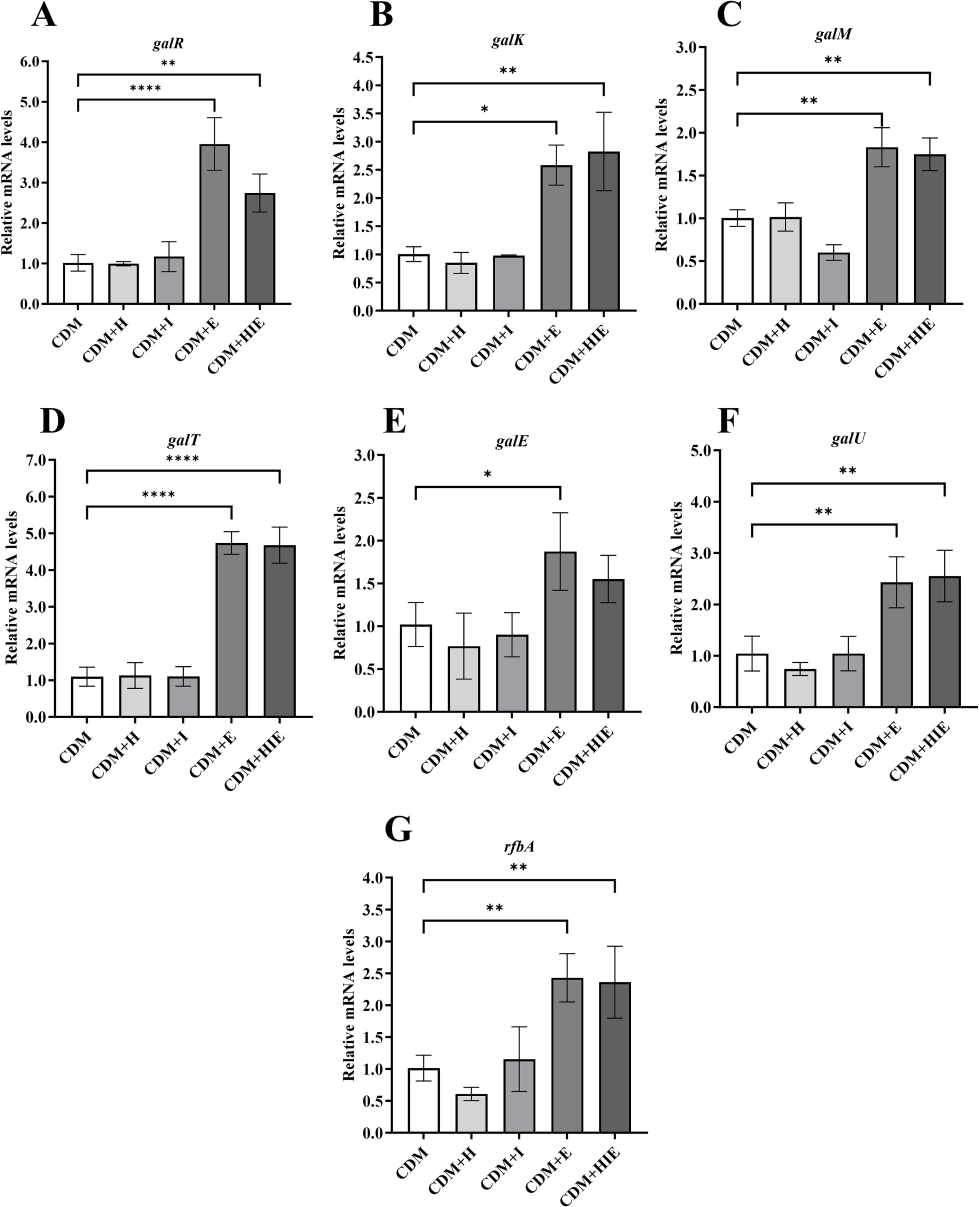
Transcript levels of galactose metabolism **(A–D)** and nucleotide sugar synthesis **(E–G)** associated genes of *Streptococcus thermophilus* 937 under different conditions. CDM + H = increasing histidine concentration to 15 mM in CDM; CDM + I = increasing isoleucine concentration to 15 mM in CDM; CDM + E = increasing glutamate concentration to 15 mM in CDM CDM + HIE = increasing histidine, isoleucine and glutamate concentrations to 15 mM in CDM. Error bars indicate SD of the mean of triplicates. Samples were analyzed using a T-test with Two-tailed calculations; *p < 0.05, **p < 0.01, ****p < 0.0001.

Compared with CDM, the transcript levels of *galK*, *galM*, *galT*, *galE* and *galU* were significantly upregulated only in CDM + E and CDM + HIE (*p* < 0.05, Figures 6B–G). This indicates that the significant upregulation of galactose metabolism-related genes of *S. thermophilus* 937 in CDM + HIE was caused by the increase in glutamate concentration. *galR* was also significantly upregulated in CDM + HIE and CDM + E (*p* < 0.05, Figure 6A), indicating that the regulatory effect of glutamate on galactose metabolism genes might be attributed to its induced expression of the *galR* transcription regulator.

### Effects of histidine or isoleucine on *epsABCD* transcript levels

**Figure 7:**
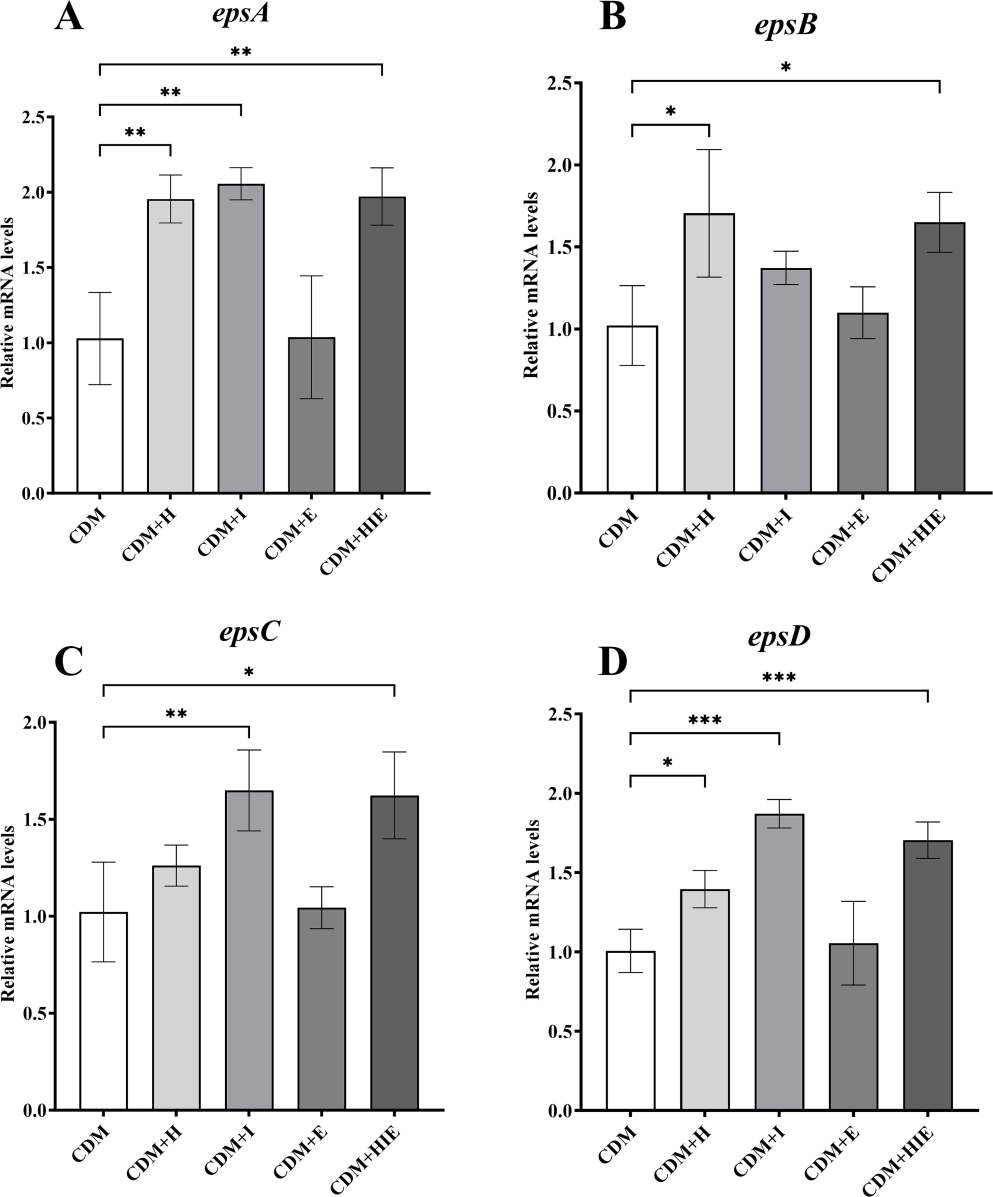
Transcript levels of genes in *epsA* **(A)**, epsB **(B)**, epsC **(C)**, and epsD **(D)** of Streptococcus thermophilus 937 under different conditions. CDM + H = increasing histidine concentration to 15 mM in CDM; CDM + I = increasing isoleucine concentration to 15 mM in CDM; CDM + E = increasing glutamate concentration to 15 mM in CDM; CDM + HIE = increasing histidine, isoleucine and glutamate concentrations to 15 mM in CDM; Error bars indicate SD of the mean of triplicate. Samples were analyzed using a T-test with Two-tailed calculations; *p < 0.05, **p < 0.01, ***p < 0.0005.

Compared with CDM, the transcriptional levels of *epsA* and *epsD* in *S. thermophilus* 937 were significantly upregulated in CDM + H, CDM + I, and CDM + HIE (*p* < 0.05, Figures 7A,D), suggesting that the increase in histidine or isoleucine concentration could upregulate the transcript levels of *epsA* and *epsD*. The transcriptional level of *epsB* was significantly upregulated in CDM + H and CDM + HIE (*p* < 0.05, Figure 7B), and the transcriptional level of *epsC* was significantly upregulated in CDM + I and CDM + HIE (*p* < 0.05, Figure 7C), indicating that the upregulation of the transcriptional levels of *epsB* and *epsC* might be caused by the increase in histidine and isoleucine concentrations, respectively.

### Effects of isoleucine or glutamate on the transcript levels of genes related to amino acid metabolism and biofilm formation

**Figure 8:**
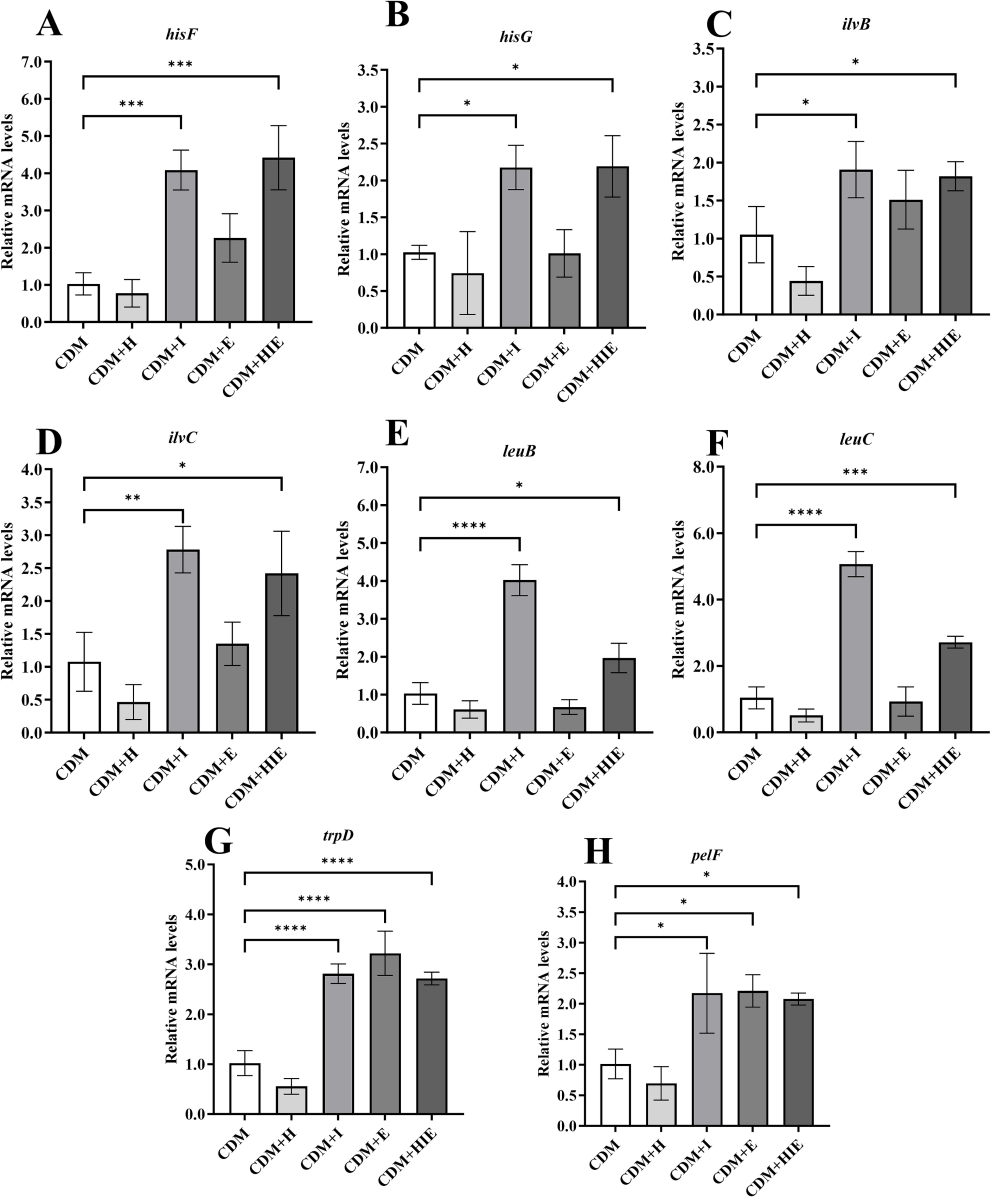
Transcript levels of amino acid metabolism **(A–F)**, purine metabolism **(G)** and biofilm formation **(H)** related genes of *Streptococcus thermophilus* 937 under different conditions. CDM + H = increasing histidine concentration to 15 mM in CDM; CDM + I = increasing isoleucine concentration to 15 mM in CDM; CDM + E = increasing glutamate concentration to 15 mM in CDM; CDM + HIE = increasing histidine, isoleucine and glutamate concentrations to 15 mM in CDM; Error bars indicate SD of the mean of triplicate. Error bars indicate SD of the mean of triplicate. Samples were analyzed using a T-test with Two-tailed calculations; *p < 0.05, **p < 0.01, ***p < 0.0005, ****p < 0.0001.

Compared with CDM, the transcriptional levels of *hisF*, *hisG*, *ilvB*, *ilvC*, *leuB* and *leuC* in *S. thermophilus* 937 were significantly upregulated in CDM + I and CDM + HIE (*p* < 0.05, Figures 8A–F), indicating that increasing the concentration of isoleucine in CDM can significantly upregulate the transcriptional levels of histidine/valine/leucine/isoleucine metabolism-related genes. Among them, *hisF* and *hisG* involved in the histidine synthesis pathway of PRPP, and their upregulation may promote the synthesis of histidine in PRPP; *IlvB*, ilvC, *leuB*, and *leuC* involved in the pyruvate synthesis of isoleucine, valine, and leucine pathways. Upregulation of their transcript levels may promote pyruvate synthesis of isoleucine, valine, and leucine. The transcriptional levels of *trpD* and *pelf* were significantly upregulated in CDM + I, CDM + E and CDM + HIE, indicating that the increase in isoleucine or glutamate concentration could upregulate the transcript levels of phenylalanine/tyrosine/tryptophan biosynthesis- and biofilm formation-related genes (*p* < 0.05, Figures 8G,H).

## Discussion

High-throughput transcriptome sequencing technology was used to compare the transcriptome characteristics of *S. thermophilus* 937 fermented in CDM and CDM + HIE at 5 h. The high-quality RNA-seq data provided sufficient depth and coverage for calculating the abundance of detected coding genes. Compared with CDM, COG annotation analysis showed that the number of differentially expressed genes involved in amino acid transport and metabolism was the largest in CDM + HIE. According to the KEGG pathway annotation results, among the significantly upregulated genes, the genes related to amino acid metabolism and carbohydrate metabolism accounted for the largest proportion.

The transcript levels of the coding gene of the enzyme involved in nucleotide sugar synthesis was compared when *S. thermophilus* 937 used lactose as a sugar in CDM or CDM + HIE. The results showed that the transcript levels of the genes related to galactose metabolism and nucleotide sugar synthesis were significantly upregulated (*p* < 0.05, CDM + HIE vs. CDM, Figure 2), which is consistent with the results of transcript levels in previous studies (Wa et al., 2022). Many studies have shown that the accumulation of nucleotide sugar can increase f-EPS production (Levander et al., 2002; Mozzi et al., 2003; Svensson et al., 2007).

Combined with the qPCR results, the upregulation of the transcript levels of genes related to galactose metabolism and nucleotide sugar synthesis was attributed to the increase in glutamate concentration (Figure 6). It is worth noting that the increase in glutamate concentration also upregulates the transcript levels of the galactose metabolism transcription regulator *galR*. Few studies have shown that the metabolic pathway of glutamate is related to the galactose pathway. Therefore, it can be speculated that the increased concentration of glutamate may induce the expression of *galR*, which upregulates the transcript levels of galactose metabolism-related genes (Vaughan et al., 2001). In *S. thermophilus*, the important function of glutamate metabolism is to produce ornithine and participate in the synthesis of arginine (Fernandez and Zuniga, 2006). In addition, glutamate, with the participation of H^+^, may produce GABA by glutamate decarboxylase (*γ*-aminobutyric acid) to increase intracellular pH (Fernandez and Zuniga, 2006). However, the *gad* encoding glutamate decarboxylase was not annotated in *S. thermophilus* 937. Milena’s statistics show that less than 10% of *S. thermophilus* can produce GABA (Brasca et al., 2016). Therefore, in *S. thermophilus* 937, glutamate may not be able to increase the intracellular pH of the strain by producing GABA.

During the logarithmic growth phase (5 h culture), the transcription levels of the *eps* gene cluster showed minimal changes between CDM + HIE and CDM conditions (Figure 3). This observation aligns with previous research suggesting that gene transcription within the *eps* cluster may be regulated by the cell growth cycle (Padmanabhan et al., 2018). Notably, prior studies have demonstrated that these three amino acids can significantly enhance the transcription of *epsABCD* genes when cells are cultured for 3 h (Wa et al., 2022). *epsA* encodes the phosphotransferase belonging to the LCP family of proteins and the transcriptional regulation of the *eps* operon in *S. thermophilus* has long been attributed to EpsA (Zeidan et al., 2017). *epsBCD* encodes the cytoplasmic domain of tyrosine protein kinase, a phosphate regulatory system controlling the assembly mechanism of *S. thermophilus* polysaccharide (Zeidan et al., 2017; Wu et al., 2022).

In this study, it was found that the transcript level of *epsAD* was regulated by histidine and isoleucine concentrations, the transcript level of *epsB* was regulated by histidine concentrations, and the transcript level of *epsC* was regulated by isoleucine concentrations according to the qPCR results (Figure 7).

In *S. thermophilus*, the main metabolite of isoleucine is *α*-oxygenic acid, which may participate in the regeneration of NAD^+^ intracellular pool, thus making more effective use of energy production pathways such as glycolysis (Fernandez and Zuniga, 2006). The two important metabolites of histidine are histidine betaine and ergometrine (2-mercapto-histidine betaine), which are commonly used by bacterial strains to maintain a reducing environment (Grigat et al., 2007; Nakajima et al., 2015; Halliwell et al., 2016), but few studies have shown the effect of histidine and isoleucine or their metabolites on the gene transcript levels of the *eps* cluster. The gene transcript levels of the *eps* cluster are affected by the growth cycle of the strain, so it can be speculated that the factors that regulate the growth of the strain may regulate the transcription of the *eps* cluster.

**Figure 9:**
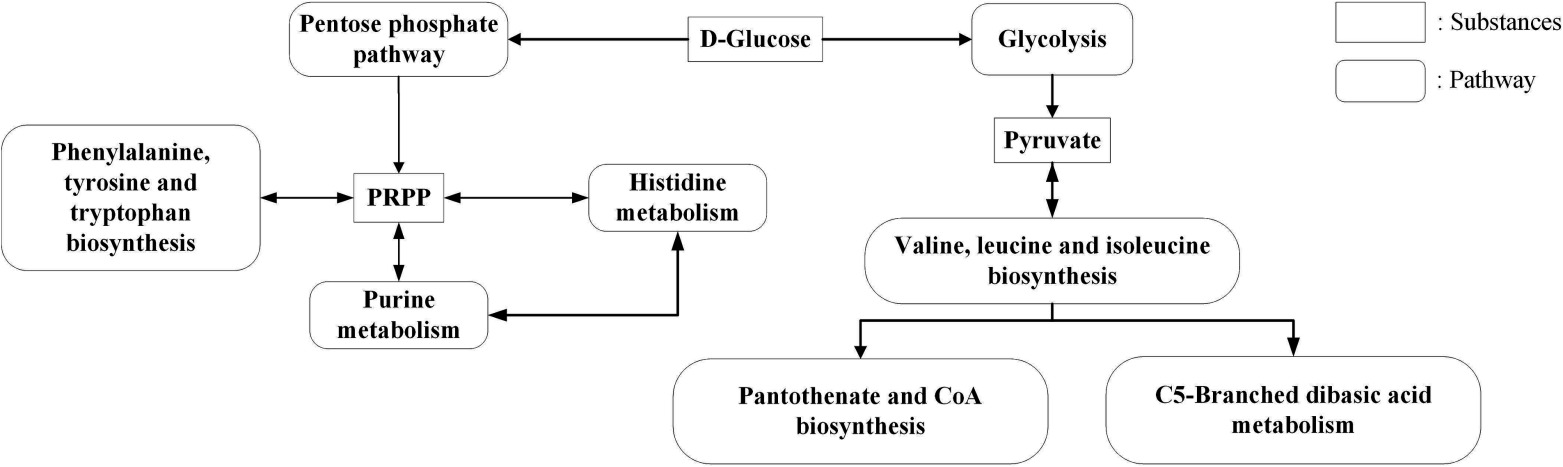
KEGG significant enrichment pathway relationship.

In KEGG enrichment analysis, 13 metabolic pathways with significant differences were enriched (Figure 4). According to the metabolic substances involved in the significantly different genes in the enriched pathways, the relationship between the metabolic pathways is sorted out as shown in Figure 9. The amino acid metabolic synthesis pathway is mainly connected with the pentose phosphate and glycolysis pathways through 5-phosphoribosyl 1-pyrophosphate (PRPP) and pyruvate. PRPP is the key metabolite of glucose metabolism in the pentose phosphate pathway. It is also the critical intermediate product involved in histidine metabolism, phenylalanine/tyrosine/tryptophan biosynthesis, and purine metabolism. In this study, after the concentration of 3 amino acids increased, the transcript levels of genes involved in PRPP metabolism in histidine metabolism, phenylalanine/tyrosine/tryptophan biosynthesis, and purine metabolism pathways were significantly upregulated (Figure 8), which may increase the consumption of PRPP and then lead to an increase in the reaction flux of Glc 6-P (glucose 6-phosphate) to produce PRPP through the pentose phosphate pathway, thereby weakening the reaction flux of Glc 6-P to produce lactic acid through the glycolysis pathway. Cohen’s research also found that the increase in the utilization rate of PRPP is of great significance for maintaining the basic life activities of cell genetics, development and growth (Cohen, 2016). Pyruvate is the key intermediate product of Glc 6-P through glycolysis to produce lactic acid, and it is also an important precursor for the synthesis of branched-chain amino acids (Fernandez and Zuniga, 2006). After the concentration of 3 amino acids increased, the transcript levels of genes in the synthesis pathway of branched-chain amino acids were significantly upregulated (Figure 8), which may promote the reaction flux of pyruvate synthesis of branched-chain amino acids, resulting in the reduction of lactic acid production. Combined with the results of previous studies (Wa et al., 2022), the change in reaction flux involving PRPP and pyruvate may be the reason why *S. thermophilus* 937 consumes more lactose, but lactic acid production remains unchanged and has a higher intracellular pH in CDM + HIE. The upregulation of gene transcript levels in the above pathway is caused by the increase in isoleucine concentration according to qPCR results (Figure 8). Studies have shown that the branched chain amino acid metabolism of *S. thermophilus* is of great significance for maintaining strain activity and intracellular pH (Garault et al., 2000). This is also the reason why *S. thermophilus* 937 also has a higher number of viable bacteria in CDM that only increases the concentration of isoleucine (Wa et al., 2022). In summary, the increase in isoleucine concentration promotes the consumption of PRPP and pyruvate and reduces lactic acid production by stimulating the upregulation of gene transcript levels in the histidine, phenylalanine, tyrosine, tryptophan, valine, leucine, and isoleucine metabolic pathways, thus maintaining the intracellular pH value and the viable cell count of *S. thermophilus* 937 and finally increasing f-EPS production.

Although it can be found that the increased concentration of isoleucine or glutamate can significantly increase the transcript levels of *pelF* and *pelG* (Figure 8), we did not see that *S. thermophilus* 937 could form a biofilm in the previous pre-experiment. According to the sequencing results of the whole genome, it is speculated that it may be because *S. thermophilus* 937 does not have a complete gene generated by the biofilm. Recent study have shown that the proteins encoded by *pelD*, *pelE*, *pelF*, and *pelG* form complexes in the inner membrane of *Pseudomonas aeruginosa*, responsible for the polymerization and cross cytoplasmic membrane transport of Pel polysaccharides (Whitfield et al., 2020). Therefore, we speculate that the specific effects caused by the elevated transcription levels of *pelF* and *pelG* genes in this study may be related to the secretion of f-EPS, but further verification is needed in subsequent experiments.

## Conclusion

In this study, transcriptome technology was used to analyze the regulatory mechanism of histidine, isoleucine and glutamate on the biosynthesis of f-EPS in *S. thermophilus* 937 at the global transcript levels, and the respective regulatory mechanism of the three amino acids was explored by qPCR. The results showed that the 3 amino acids had different mechanisms to enhance the biosynthesis of f-EPS of *S. thermophilus* 937. The increase in isoleucine concentration upregulated the transcriptional levels of genes related to histidine and branched-chain amino acid synthesis, thus promoting the consumption of PRPP and pyruvate and finally maintaining the viable cell count. The increase in glutamate concentration promoted galactose metabolism and nucleotide sugar synthesis. The increase in histidine concentration increased the transcript levels of *epsABD*. This study further clarified the regulatory effects of histidine, isoleucine, and glutamate on the biosynthesis of f-EPS and provided a new idea for regulating the biosynthesis of f-EPS in *S. thermophilus*.

## Data Availability

The data presented in the study are deposited in the China National Center for Bioinformation (CNCB) repository, accession number CRA019170.

## Footnotes

^1^ www.majorbio.com

## References

[ref1] Brasca M. Hogenboom J. A. Morandi S. Rosi V. D'Incecco P. Silvetti T.. (2016). Proteolytic activity and production of γ-aminobutyric acid by *Streptococcus thermophilus* cultivated in microfiltered pasteurized milk. Agric. Food Chem. 64, 8604–8614. doi: 10.1021/acs.jafc.6b03403, PMID: 27787997

[ref2] Chen Y. Zhang M. Ren F. (2019). A role of exopolysaccharide produced by *Streptococcus thermophilus* in the intestinal lnflammation and mucosal barrier in Caco-2 monolayer and dextran sulphate sodium-induced experimental murine colitis. Molecules 24, 1–13. doi: 10.3390/molecules24030513, PMID: 30708992 PMC6384629

[ref3] Cohen G. N. (2016). The biosynthesis of histidine and its regulation. New York, NY: Springer.

[ref4] Fernandez M. Zuniga M. (2006). Amino acid catabolic pathways of lactic acid bacteria. Crit. Rev. Microbiol. 32, 155–183. doi: 10.1080/10408410600880643, PMID: 16893752

[ref5] Garault P. Letort C. Juillard V. Monnet V. J. A. Microbiology E. (2000). Branched-chain amino acid biosynthesis is essential for optimal growth of *Streptococcus thermophilus* in milk. Appl. Environ. Microb. 66, 5128–5133. doi: 10.1128/AEM.66.12.5128-5133.2000, PMID: 11097879 PMC92433

[ref6] Grigat S. Harlfinger S. Pal S. Striebinger R. Golz S. Geerts A.. (2007). Probing the substrate specificity of the ergothioneine transporter with methimazole, hercynine, and organic cations. Biochem. Pharmacol. 74, 309–316. doi: 10.1016/j.bcp.2007.04.015, PMID: 17532304

[ref7] Halliwell B. Cheah I. K. Drum C. L. J. B. Communications B. R. (2016). Ergothioneine, an adaptive antioxidant for the protection of injured tissues? A hypothesis. Biochem. Bioph. Res. Co. 470, 245–250. doi: 10.1016/j.bbrc.2015.12.124, PMID: 26772879

[ref8] Iyer R. Tomar S. K. Maheswari T. U. Singh R. (2010). *Streptococcus thermophilus* strains: multifunctional lactic acid bacteria. Int. Dairy J. 20, 133–141. doi: 10.1016/j.idairyj.2009.10.005

[ref9] Letort C. Juillard V. (2001). Development of a minimal chemically-defined medium for the exponential growth of *Streptococcus thermophilus*. J. Appl. Microbiol. 91, 1023–1029. doi: 10.1046/j.1365-2672.2001.01469.x, PMID: 11851809

[ref10] Levander F. Svensson M. Radstrom P. (2002). Enhanced exopolysaccharide production by metabolic engineering of *Streptococcus thermophilus*. Appl. Environ. Microbiol. 68, 784–790. doi: 10.1128/AEM.68.2.784-790.2002, PMID: 11823219 PMC126717

[ref11] Li C. Li W. Chen X. Feng M. Rui X. Jiang M.. (2014). Microbiological, physicochemical and rheological properties of fermented soymilk produced with exopolysaccharide (EPS) producing lactic acid bacteria strains. LWT 57, 477–485. doi: 10.1016/j.lwt.2014.02.025

[ref12] Livak K. J. Schmittgen T. D. (2001). Analysis of relative gene expression data using real-time quantitative PCR and the 2^-ΔΔCT^ method. Methods 25, 402–408. doi: 10.1006/meth.2001.1262, PMID: 11846609

[ref13] Merrick M. Edwards R. A. (1995). Nitrogen control in bacteria. Microbiol. Rev. 59, 604–622. doi: 10.1128/mr.59.4.604-622.1995, PMID: 8531888 PMC239390

[ref14] Mizuno H. Tomotsune K. Islam M. A. Funabashi R. Albarracin L. Ikeda-Ohtsubo W.. (2020). Exopolysaccharides from *Streptococcus thermophilus* ST538 modulate the antiviral innate immune response in porcine intestinal epitheliocytes. Front. Microbiol. 11:894. doi: 10.3389/fmicb.2020.00894, PMID: 32508770 PMC7248278

[ref15] Mozzi F. Savoy de Giori G. de Valdez F. (2003). UDP-galactose 4-epimerase: a key enzyme in exopolysaccharide formation by *Lactobacillus casei* CRL 87 in controlled pH batch cultures. J. Appl. Microbiol. 94, 175–183. doi: 10.1046/j.1365-2672.2003.01821.x 12534808

[ref16] Nakajima S. Satoh Y. Yanashima K. Matsui T. Dairi T. (2015). Ergothioneine protects *Streptomyces coelicolor* A3(2) from oxidative stresses. J. Biosci. Bloeng. 120, 294–298. doi: 10.1016/j.jbiosc.2015.01.013, PMID: 25683449

[ref17] Padmanabhan A. Tong Y. Wu Q. Zhang J. Shah N. P. (2018). Transcriptomic insights into the growth phase- and sugar-associated changes in the exopolysaccharide production of a high EPS-producing *Streptococcus thermophilus* ASCC 1275. Front. Microbiol. 9:1919. doi: 10.3389/fmicb.2018.01919 30177921 PMC6109772

[ref18] Papadimitriou K. Alegria A. Bron P. A. de Angelis M. Gobbetti M. Kleerebezem M.. (2016). Stress physiology of lactic acid bacteria. Microbiol. Mol. Biol. Rev. 80, 837–890. doi: 10.1128/MMBR.00076-15, PMID: 27466284 PMC4981675

[ref19] Svensson M. Lohmeier-Vogel E. Waak E. Svensson U. Radstrom P. (2007). Altered nucleotide sugar metabolism in *Streptococcus thermophilus* interferes with nitrogen metabolism. Int. J. Food Microbiol. 113, 195–200. doi: 10.1016/j.ijfoodmicro.2006.06.032, PMID: 16996629

[ref20] Vaughan E. E. van den Bogaard P. T. Catzeddu P. Kuipers O. P. de Vos W. M. (2001). Activation of silent gal genes in the lac-gal regulon of *Streptococcus thermophilus*. J. Bacteriol. 183, 1184–1194. doi: 10.1128/JB.183.4.1184-1194.2001, PMID: 11157930 PMC94991

[ref21] Wa Y. Zhang C. Sun G. Qu H. Chen D. Huang Y.. (2022). Effect of amino acids on free exopolysaccharide biosynthesis by *Streptococcus thermophilus* 937 in chemically defined medium. J. Dairy Sci. 105, 6460–6468. doi: 10.3168/jds.2022-21814, PMID: 35691747

[ref22] Wa Y. Zhao X. Peng K. Qu H. Chen D. Zhang C.. (2023). Effects of nutrients on the growth of and free exopolysaccharide biosynthesis by *Streptococcus thermophilus* 937 in a chemically defined medium. Curr. Microbiol. 80:331. doi: 10.1007/s00284-023-03421-x, PMID: 37634211

[ref23] Whitfield G. B. Marmont L. S. Ostaszewski A. Rich J. D. Whitney J. C. Parsek M. R.. (2020). Pel polysaccharide biosynthesis requires an inner membrane complex comprised of PelD, PelE, PelF, and PelG. J. Bacteriol. 202:e00684-19. doi: 10.1128/jb.00684-19, PMID: 31988082 PMC7099134

[ref24] Wu J. Han X. Ye M. Li Y. Wang X. Zhong Q. (2022). Exopolysaccharides synthesized by lactic acid bacteria: biosynthesis pathway, structure-function relationship, structural modification and applicability. Crit. Rev. Food Sci. 63, 7043–7064. doi: 10.1080/10408398.2022.2043822, PMID: 35213280

[ref25] Xiong Z. Q. Kong L. H. Lai P. F. Xia Y. J. Liu J. C. Li Q. Y.. (2019). Genomic and phenotypic analyses of exopolysaccharide biosynthesis in *Streptococcus thermophilus* S-3. J. Dairy Sci. 102, 4925–4934. doi: 10.3168/jds.2018-15572, PMID: 30928267

[ref26] Zeidan A. A. Poulsen V. K. Janzen T. Buldo P. Derkx P. M. F. Oregaard G.. (2017). Polysaccharide production by lactic acid bacteria: from genes to industrial applications. FEMS Microbiol. Rev. 41, S168–S200. doi: 10.1093/femsre/fux017 28830087

